# Person-centred HIV prevention services in sub-Saharan Africa: a scoping review

**DOI:** 10.1186/s12981-025-00839-0

**Published:** 2026-02-05

**Authors:** Daniel Asogun, Augustina Konadu Larbi-Ampofo, Jesu-Oboh Akhaine, Matthew Ayomide Abiodun, Precious Enotiuwa, Paul Aikhenomian, Perpetual Osemengbe Idialu, Mark Bimbola Ekhabafe, Ivie Blessing Okhihan

**Affiliations:** 1 Department of Urology, Sheffield NHS Foundation Trust, Sheffield, UK; 2 Acute Medical Unit, Epsom and St Helier University Hospitals NHS Trust, London, UK; 3 Department of Internal Medicine, Central Hospital, Benin City, Edo State Nigeria; 4 Department of Medicine and Surgery, Ambrose Alli University, Ekpoma, Edo State Nigeria; 5 Department of Internal Medicine, Irrua Specialist Teaching Hospital, Irrua, Edo State Nigeria; 6 Department of Internal Medicine, Obafemi Awolowo University Teaching Hospital Complex, Ile-Ife, Osun State Nigeria

**Keywords:** HIV infections/prevention & control, Person-centred care, Health services accessibility, Sub-Saharan africa, Sexual and reproductive health services

## Abstract

**Background:**

In spite of numerous advancements in HIV prevention, there are still gaps that impede reaching key populations throughout sub-Saharan Africa. Traditional approaches usually overlook personal preferences, social context and structural obstacles, often leading to subpar service uptake. Person-centred care (PCC) has emerged in recent times as an approach that shows promise; however, its implementation is still not widespread in sub-Saharan Africa.

**Objective:**

This scoping review aimed to systematically map the landscape of Person- Centred HIV prevention services in Sub-Saharan Africa, identifying intervention models, outcomes, and implementation barriers and facilitators.

**Methods:**

A comprehensive search of PubMed, ScienceDirect, Google Scholar, and AJOL identified studies published between 2010 and 2025. Following PRISMA-ScR guidance, 174 records were identified, 33 duplicates removed, and 141 records screened. A total of 128 records were excluded, 13 full texts were sought, and 12 studies from six countries met the inclusion criteria. Data were charted and synthesised narratively.

**Results:**

Services included PrEP programmes, choice based models, peer-led outreach initiatives, and differentiated ART delivery systems. Essential elements of PCC included shared decision-making, decentralisation, social support, and counselling that takes into account stigma. Models that are community-based and peer-supported improved accessibility, trust, and adherence to treatment. Interventions that provided options for prevention methods and service locations consistently demonstrated improved uptake, satisfaction, and clinical outcomes. Nonetheless, implementation varied by region, with no representation from West and Central Africa and notable disparities in reach among adolescent boys, older adults, and sexual minorities.

**Conclusion:**

Evidence from the included studies indicates that person-centred strategies can enhance engagement with HIV prevention services in several settings across sub-Saharan Africa. However, the benefits were not uniform, and gaps in geographical representation, equity, and integration of broader psychosocial needs persist. Future programmes should address structural barriers, strengthen psychological safety and community trust, and ensure more inclusive design to improve the reach and consistency of person-centred HIV prevention.

**Supplementary Information:**

The online version contains supplementary material available at 10.1186/s12981-025-00839-0.

## Background

Africa continues to carry the greatest share of the global Human Immunodeficiency Virus (HIV) burden, with an estimated 26.3 million people living with the virus across the continent. By 2024, antiretroviral therapy (ART) coverage had reached approximately 84% in Eastern and Southern Africa, and 76% in Western and Central Africa—reflecting substantial progress in treatment access [1]. Globally, new HIV infections declined by 39% between 2010 and 2023, with sub-Saharan Africa leading this trend through a remarkable 56% reduction, underscoring the region’s strides in combating the epidemic [2]. Despite these gains, critical gaps in HIV prevention persist especially among adolescents and key populations. These challenges are often compounded by entrenched structural and social barriers, including stigma, gender-based inequities, criminalisation of certain groups, and chronically under-resourced health systems [3, 4].

Traditional HIV prevention initiatives in sub-Saharan Africa have focused on biomedical strategies such as HIV testing, condom usage, and oral pre-exposure prophylaxis (PrEP). However, many of these initiatives have adhered to standardised, provider-centric models that inadequately address the preferences, beliefs, or psychosocial contexts of the targeted individuals. Consequently, the uptake and adherence to prevention tools like PrEP remain sub-optimal in numerous settings [5, 6]. Additionally, the stigma associated with HIV—both social and internalised—continues to obstruct individuals from seeking preventive care or disclosing their status, further complicating early diagnosis and service utilisation [7, 8].

Brault opined that a collaborative strategy involving people, policymakers, healthcare providers, and community organisations is essential for creating a supportive atmosphere that fosters health client involvement and improves outcomes in HIV care [9]. Correspondingly, global HIV policies and programmatic frameworks have increasingly underscored the necessity for person-centred care (PCC) approaches. The World Health Organization defines PCC as care strategies and practices that regard the individual as a whole, encompassing various levels of needs and objectives, which arise from their unique social determinants of health [10]. In the context of HIV prevention, this entails the integration of services that are customised to the social and behavioural circumstances of individuals, enhancing the accessibility and acceptability of interventions, and empowering people to make informed health decisions [11]. The United States President’s Emergency Plan for AIDS Relief (PEPFAR) has likewise recognised PCC as a core principle for its programming, promoting differentiated service delivery models that incorporate individual feedback and lived experiences [12]. Recent developments in biomedical prevention tools have further emphasised the significance of PCC. Long-acting injectable PrEP (e.g. cabotegravir), monthly vaginal rings (e.g., dapivirine), and HIV self-testing are broadening the array of options available to at-risk individuals. These interventions provide greater autonomy, decrease the frequency of visits, and align more closely with individual lifestyles, presenting promising opportunities to mitigate barriers associated with stigma and the burden of care [13–15]. Mobile health (mHealth) interventions such as SMS reminders, telehealth consultations, and digital adherence monitoring have also shown potential to enhance engagement in HIV prevention services while being tailored to individual needs [16].

Moreover, strategies such as peer navigation, community-led outreach, and integration of mental health or sexual and reproductive health services into HIV programmes are consistent with PCC principles. These strategies have shown improvements in satisfaction, engagement, and stigma reduction in studies across sub-Saharan Africa and other low- and middle-income countries [17, 18]. Despite growing evidence and global policy support, the application of PCC in HIV prevention remains poorly defined and unevenly implemented. Much of the literature and programming to date has focused on PCC in treatment settings, with limited attention to how it is conceptualised, operationalised, and evaluated in prevention services [11].

This scoping review aims to address this gap by systematically mapping how PCC is applied in HIV prevention programmes in sub-Saharan Africa. It will explore how PCC principles have been integrated into prevention strategies, assess the types of service models and outcomes associated with person-centred models, and identify facilitators and barriers to effective implementation. The findings will contribute to a clearer understanding of how PCC can be leveraged to strengthen HIV prevention efforts and promote equitable, person-responsive care across the region.

## Methodology

### Study design

This scoping review was conducted following the methodological guidelines outlined in the Joanna Briggs Institute Manual for Evidence synthesis [19], which is specifically recommended for scoping reviews. The review also adheres to the Preferred Reporting Items for Systematic Reviews and Meta-Analyses extension for Scoping Reviews (PRISMA-ScR) guidelines [20]. Additionally, we employed the PCC Framework—Population, Concept, and Context - to formulate our eligibility criteria and direct the study selection process. Consistent with the objectives of a scoping review and the methodological guidance of the Joanna Briggs Institute (JBI), no formal quality appraisal of included studies was undertaken as quality assessment was deemed not appropriate for this review.

#### Inclusion

i. Population: Individuals at risk of HIV or living with HIV in Sub-Saharan Africa, including key populations (e.g., adolescents, sex workers, men having sex with men (MSM), pregnant women).
ii. Concept: Studies describing or evaluating Person- Centred HIV prevention services or models (e.g. PrEP programmes, choice based models, differentiated care, community-led services) and Treatment focused studies with PCC elements. Only peer-reviewed studies published in indexed journals were included. Grey literature was not systematically searched or assessed for eligibility.
iii. Context: Studies conducted in healthcare or community settings across Sub-Saharan Africa (e.g., primary care clinics, mobile units, outreach programs).

#### Exclusion

i. Population: Studies not focused on HIV-affected or at-risk populations.
ii. Concept: Studies not incorporating a Person- Centred approach or not focused on individual- or community-level engagement.
iii. Context: Studies conducted outside of Sub-Saharan Africa.

### Search strategy

A comprehensive literature search was conducted across multiple databases to identify relevant studies on person-centred HIV prevention services in sub-Saharan Africa. The databases searched included: PubMed, Google Scholar, ScienceDirect, and African Journals Online (AJOL). The search strategy incorporated a combination of Medical Subject Headings (MeSH), free-text keywords, and Boolean operators (AND, OR) to maximise coverage. No language restrictions were applied, and the search spanned publications from 2010 to 2025 to reflect contemporary practices in HIV prevention. Furthermore, although no language restrictions were applied, we recognise that the search was implemented primarily with English keywords and in databases with predominantly Anglophone coverage. This may have introduced functional language bias despite the absence of formal language limits. To make this explicit and aid reproducibility, we provide French and Portuguese free-text equivalents of our core concepts (HIV prevention; person-centred care; sub-Saharan Africa) in the Appendix, which future replications can apply to French- and Portuguese-language databases and indices. We also note that our original strategy did not systematically include grey literature sources, which may host community-led innovations from West and Central Africa that are not indexed in bibliographic databases. The search strategy is contained in Table 1.

**Table 1:** Expanded and detailed search strategy

Database	Search Strategy	Hits
PubMed	(“HIV prevention“[MeSH Terms] OR “HIV prevention services“[All Fields] OR (“HIV“[All Fields] AND “prevention“[All Fields])) AND (“patient-centred care“[MeSH Terms] OR “person-centred“[All Fields] OR “client-centred“[All Fields] OR “patient focused“[All Fields]) AND (“Africa South of the Sahara“[MeSH Terms] OR “sub-Saharan Africa“[All Fields])	22
ScienceDirect	(“HIV prevention” AND (“person-centred” OR “client-centred”)) AND (“sub-Saharan Africa”)	118
AJOL	(“HIV prevention” AND “person-centred”) AND (“sub-Saharan Africa”)	9
Google Scholar	(“HIV prevention services” AND “person-centred care” AND “sub-Saharan Africa”)	25

### Selection of studies

Studies retrieved were first screened by title and abstract by three independent reviewers to remove duplicates and irrelevant articles. Full-text screening followed for potentially eligible studies. Grey literature was not included due to difficulties in retrieval and verification. We acknowledge that this decision may disproportionately exclude reports from community-based organisations and implementers in West and Central Africa, where programme documentation is frequently disseminated outside indexed journals. Discrepancies in article selection were resolved by discussion among reviewers to achieve consensus. Studies meeting the inclusion criteria were advanced for data extraction.

### Data extraction and management

A standardised data extraction form was developed using Google Sheets. Key information collected included: author(s), publication year, country of study, study design, population characteristics, description of the person-centred HIV prevention intervention, person-centred features, setting of delivery, outcomes measured (e.g. uptake, satisfaction), barriers, and facilitators.

### Data synthesis

Extracted data were synthesised using a narrative approach, organising results by population type, intervention modality, delivery setting, and outcomes. For this review, we adopted the Institute of Medicine (IOM) [21] framing of person-centred care as care that is respectful of and responsive to individual preferences, needs, and values, ensuring that their values guide clinical decisions. We considered models to be PCC-aligned if they explicitly incorporated at least one of the following features: Shared decision-making or co-design of services; Individual tailoring of prevention packages; Respect for client autonomy and preferences (e.g., choice of prevention method, modality, or setting); Holistic or integrated care responsive to broader psychosocial needs while involving family and friends. Models that described only decentralisation (e.g., shifting care from hospitals to primary centres) or community-based delivery without explicit or implicit reference to client involvement, tailoring, or autonomy were not involved in this study. When features were implicit, we applied the IOM domains and our framework to the study descriptions. In cases of ambiguity, two reviewers independently reviewed the text, and discrepancies were resolved by discussion until consensus was reached. Tabular summaries were used to present study characteristics and findings. A geographic map was also used to depict the distribution of studies across sub-Saharan Africa and a conceptual diagram shows the relationship between PCC components and outcomes.

### Thematic analysis

To identify recurring patterns in person-centred HIV prevention service models, we conducted a deductive thematic analysis guided by established PCC frameworks from the Institute of Medicine (IOM) [21]. A structured coding matrix was developed based on predefined domains of PCC (e.g., shared decision-making, autonomy, accessibility etc.). Two reviewers independently applied this matrix to the extracted data, identifying key person centered features across studies. Discrepancies were discussed and resolved until consensus was reached. Thematic saturation was achieved when no new categories emerged from the final set of studies. Themes were then synthesised narratively and tabulated in the Results section. We did not extract verbatim participant quotes, as our aim was to provide a thematic overview of person-centred approaches rather than a qualitative meta-synthesis.

**Fig. 1:**
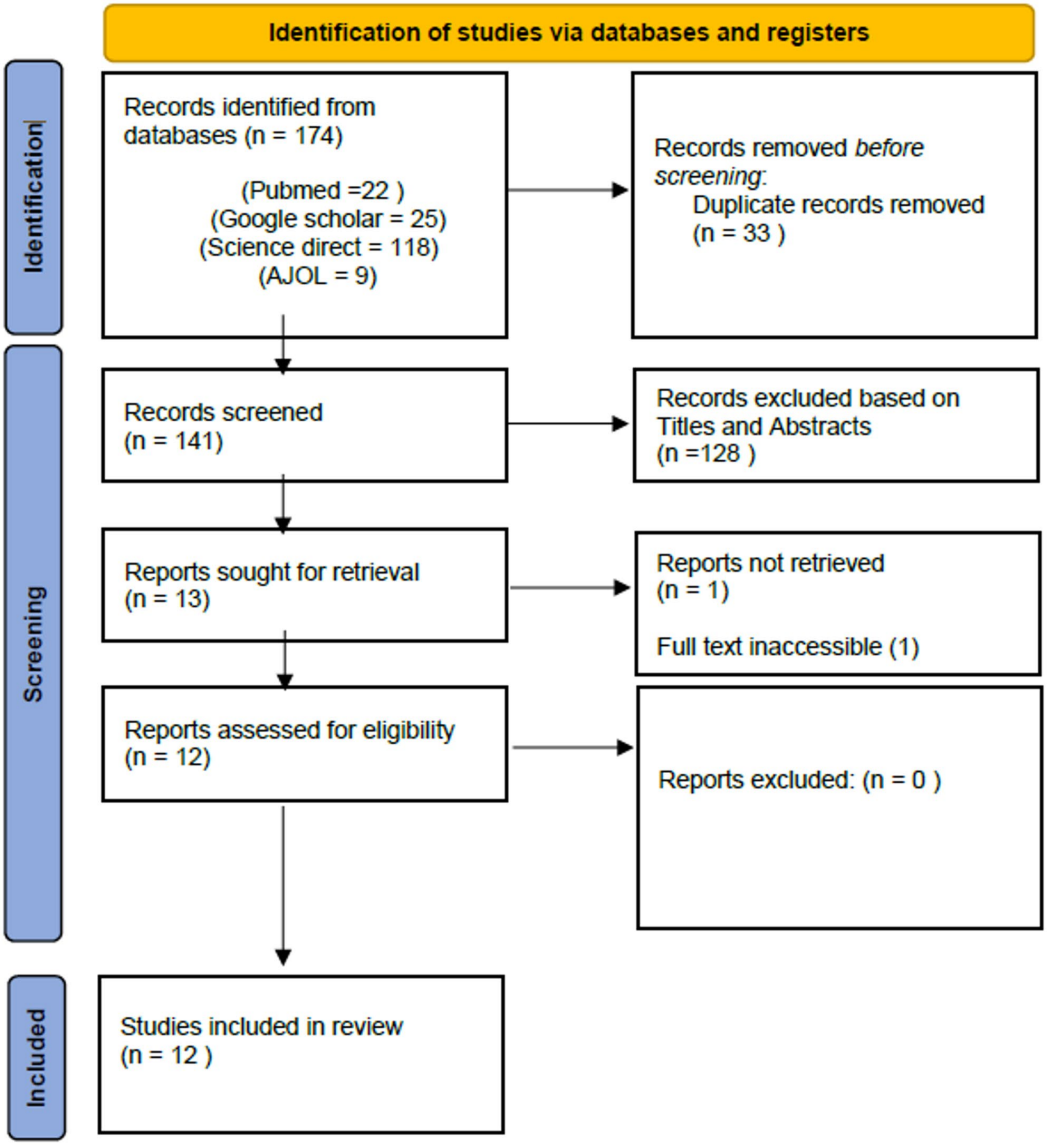
PRISMA chart. (Source: Page MJ, et al. BMJ 2021;372:n71. 10.1136/bmj.n71)

## Results

### Study selection

A total of 12 studies were included after screening 174 records, removing 33 duplicates, and excluding 129 studies that did not meet the inclusion criteria. One study could not be retrieved. The PRISMA flow diagram (Fig. 1) illustrates the selection process.

**Table 2:** Table showing the selected papers and study characteristics

	Authors	Study title	Publication year	Country	Study design	Study population	Sample size	Study setting^#^
1	Omollo et al. [22]	Provider–client rapport in pre-exposure prophylaxis delivery: a qualitative analysis of provider and client experiences of an implementation science project in Kenya	2022	Kenya	Qualitative study using in-depth, semi-structured interviews and thematic content analysis	Adolescent girls and young women (AGYW) between 16–25 years, and healthcare providers (HCPs)	53 participants (38 AGYW + 15 HCPs)	Facility-based
2	Koss et al. [23]	Dynamic choice HIV prevention intervention at outpatient departments in rural Kenya and Uganda: a randomized trial	2023	Kenya and Uganda	Randomised control trial	Individuals aged ≥15 years with current or anticipated HIV exposure risk (male & female)	403 participants	Facility-based and community-based
3	Koss et al. [24]	HIV incidence after pre-exposure prophylaxis initiation among women and men at elevated HIV risk: a population-based study in rural Kenya and Uganda	2021	Kenya and Uganda	Population-based study	Individuals aged ≥15 years at elevated HIV risk (male & female)	5,447 PrEP initiators	Community-based
4	Mataboge et al. [25]	Planning for decentralized, simplified PrEP: Learnings from potential end users in Ga-Rankuwa, Gauteng, South Africa	2023	South Africa	Qualitative study using Focused group discussions and inductive thematic analysis	AGYW, pregnant AGYW, female sex workers, adolescent boys and young men (ABYM), and men who have sex with men (MSM)	109 participants	Facility-based and community-based
5	Williams et al. [26]	Implementation of the Automated Medication Dispensing System–Early Lessons From Eswatini	2023	Eswatini	Implementation science study	Clinically stable adult ART clients living with HIV aged ≥ 18 years (male & female)	Not specified	Facility-based
6	Kabami et al. [27]	Uptake of a patient-centred dynamic choice model for HIV prevention in rural Kenya and Uganda: SEARCH SAPPHIRE study	2023	Kenya and Uganda	Randomised control trial	Individuals at elevated risk of HIV, including pregnant women, out patient department (OPD) patients, and community members, aged ≥ 15years (male & female)	612 participants	Facility-based and community-based
7	Kakande et al. [28]	A Community-Based Dynamic Choice Model for HIV Prevention Improves PrEP and PEP Coverage in Rural Uganda and Kenya: a Cluster Randomised Trial	2023	Kenya and Uganda	Randomised control trial	Individuals at risk of HIV aged ≥ 15years (male & female)	429 participants	Community-based
8	Jani et al. [29]	Reducing HIV-related risk and mental health problems through a client-centred psychosocial intervention for vulnerable adolescents in Addis Ababa, Ethiopia	2016	Ethiopia	Quasi-experimental pre-post Cohort study	Migrant adolescents aged 15–18 years	730 participants	Community-based
9	Zewdie et al. [30]	Effect of differentiated direct-to-pharmacy PrEP refill visits supported with client HIV self-testing on clinic visit time and early PrEP continuation	2024	Kenya	Quasi-experimental study	Clients aged ≥ 18 years receiving PrEP at four participating clinics	746 clients	Facility-based
10	Nkolo et al. [31]	Clients in Uganda accessing preferred differentiated antiretroviral therapy models achieve higher viral suppression and are less likely to miss appointments: a cross-sectional analysis	2023	Uganda	Quantitative study	Clients aged 1–92 years receiving ART at selected health facilities	6376 clients	Facility-based and community-based
11	Kamya et al. [32]	Dynamic choice HIV prevention with cabotegravir long-acting injectable in rural Uganda and Kenya: a randomised trial extension	2024	Kenya and Uganda	Randomised trial extension	Individuals aged ≥ 15 years at risk for HIV from antenatal, outpatient, and community settings	984 participants	Facility-based and community-based
12	Cowan et al. [33]	A risk-differentiated, community-led intervention to strengthen uptake and engagement with HIV prevention and care cascades among female sex workers in Zimbabwe (AMETHIST): a cluster randomised trial	2024	Zimbabwe	Randomised control trial	Cisgender female sex workers aged ≥ 18 years	4268 female sex workers	Facility-based and community-based

^#^Setting definitions: Facility-based = clinic, hospital or primary care centre; Community-based = mobile unit, outreach venue (e.g., school, recreational space), pharmacy, participants’ homes, community drug distribution point, or peer-led support group

### Characteristics of included studies

**Fig. 2:**
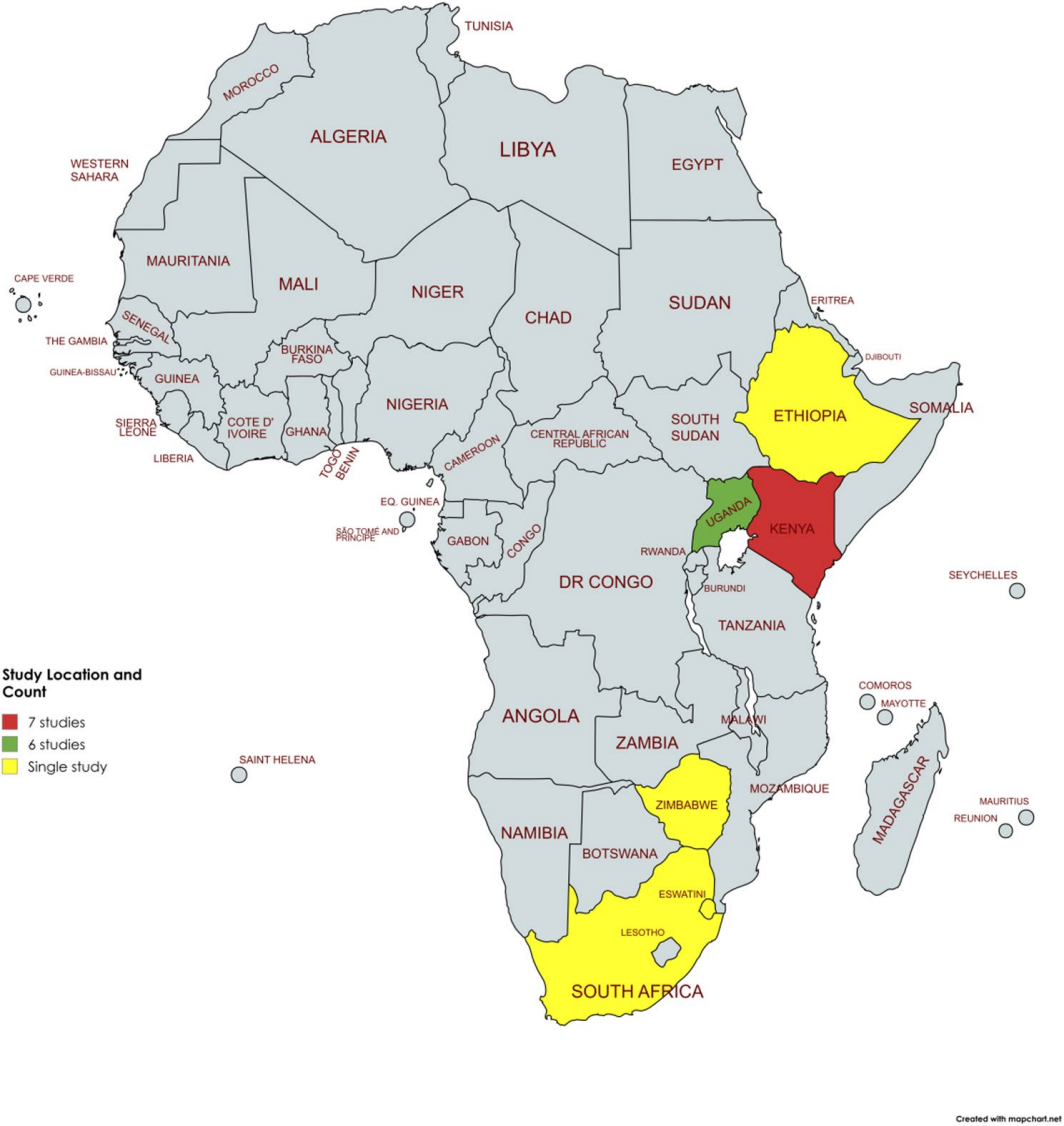
A map depicting location of studies in Sub-Saharan Africa

The studies included in this review (*n* = 12) were published between 2016 and 2024, with a notable concentration in 2023 (six studies) [23, 25–28, 31]. These 12 studies were carried out in six different countries: Kenya, Uganda, Ethiopia, Zimbabwe, South Africa, and Eswatini, encompassing both facility-based and community-based interventions for HIV prevention and treatment (refer to Fig. 2). All included studies were from Eastern and Southern Africa; no studies from West or Central Africa met inclusion. We explicitly flag this as a regional evidence gap in the mapped literature rather than an absence of activity in those settings. The designs of the studies were diverse, comprising randomised control trials (*n* = 5) [23, 27, 28, 32, 33], qualitative studies (*n* = 2) [22, 25], quasi-experimental studies (*n* = 2) [29, 30], quantitative studies (*n* = 2) [29, 31], population-based studies (*n* = 1) [24], and implementation science studies (*n* = 1) [26]. These various methodologies were employed to evaluate the effectiveness, accessibility, and acceptability of different HIV prevention strategies. In terms of population characteristics, the studies involved adolescent girls and young women (AGYW), female sex workers, individuals at current or anticipated risk of HIV exposure, and the general population utilizing HIV services. Participants’ ages were predominantly ≥ 15 years including both male and female individuals, however one study enrolled clients aged 1 to 92 years, encompassing both paediatric and adult populations [31]. Certain studies specifically targeted key populations, such as sex workers, men who have sex with men (MSM), pregnant AGYW, and those undergoing antiretroviral therapy (ART) therapy. Sample sizes varied significantly among the studies, with some focusing on small groups for qualitative insights—such as a study in Kenya involving 53 participants while others included larger cohorts, like 6376 ART clients in a study conducted in Uganda. The cumulative sample size across all studies was over 20,157 individuals. The study settings were classified as facility-based, community-based, or both. Facility-based referred to services delivered in formal health facilities like clinics or hospitals [22, 26, 30]. Community-based included non-clinical venues such as pharmacies, schools, mobile units, home visits, and peer-led outreach [24, 28, 29]. A total of six studies utilised a combination of facility-based and community delivery methods, thereby broadening the options available for individuals seeking HIV prevention services [23, 25, 27, 31–33]. This is all summarised in Table 2.

### Characteristics of HIV prevention services

Person-centred HIV prevention services encompassed PrEP delivery (9 studies) [22–25, 27, 28, 30, 32, 33], HIV testing and counselling (8 studies) [22–24, 27, 28, 30, 32, 33], the Dynamic Choice HIV Prevention (DCP) Model (4 studies) [23, 27, 28, 32], psychosocial interventions (1 study) [29], differentiated ART delivery model (1 study) [31], a risk-differentiated, peer-led HIV prevention and care model (1 study) [33], and automated medication dispensing (1 study) [26]. Various tailored service delivery mechanisms were investigated, including self-testing, the establishment of provider-client rapport, flexible medication refills, empowerment through peer-led support groups, and personalised prevention options. The key findings underscore notable enhancements in PrEP adherence, the uptake of HIV prevention strategies, and viral suppression when interventions integrated client choice, decentralised services, and structured counselling (See Table 3).

**Table 3:** Table showing person-centred characteristics, barriers and facilitators

	Authors	Type of service delivery	Components of service delivery	Description of person-centred characteristics	Outcomes/key findings/insights	Barriers	Facilitators	Degree of person centered care alignment
1	Omollo et al. [22]	Pre-exposure prophylaxis (PrEP) programme	Pre-exposure prophylaxis (PrEP)	• Use of friendly and non-judgemental tones • Maintenance of confidentiality to build client trust • Conversational approaches for effective risk assessment • Tailored counselling to enhance client knowledge and self-efficacy • Empowerment strategies that support client agency	• Importance of strong provider–client rapport for effective PrEP uptake and continuation among AGYW • Five key strategies used by healthcare providers to build rapport included friendly communication, confidentiality, conversational counselling, active listening, and supporting client decision-making • Positive provider interactions facilitated higher adherence to PrEP, while negative experiences discouraged AGYW from continuing its use • Incorporating these relationship-building strategies into PrEP provider training may improve HIV prevention outcomes	• Pre-existing societal stigma against young unmarried women being sexually active • Fear among AGYW of being judged when seeking reproductive health services • Concerns about privacy and confidentiality when discussing personal risk factors	• Youth-friendly services • Supportive, non-judgemental counselling • Tailored information addressing individual needs • Trust-building strategies • Empowerment of AGYW to make informed decisions	Explicitly aligned
2	Koss et al. [23]	Dynamic Choice HIV Prevention (DCP) model^$^	Pre-exposure prophylaxis (PrEP) and post-exposure prophylaxis (PEP)	• Provider training on person-centred service delivery • Choice-based decision-making • Structured counselling approaches • Psychological support for trauma	• Intervention participants in the DCP arm had significantly higher biomedical HIV prevention coverage than those in standard-of-care (SOC) • 86% of DCP participants chose PrEP, and 15% chose PEP, with many switching preferences over time • Self-reported PrEP/PEP coverage was 47.5% in DCP vs. 18.3% in SOC, indicating two-fold higher engagement in prevention • When restricting analysis to periods of self-reported HIV exposure risk, DCP participants had 64.9% prevention coverage vs. 26.3% in SOC, demonstrating greater adherence during high-risk periods	• Low PrEP uptake due to societal stigma • Hesitation around daily pill-taking commitment • Long wait times for HIV services, discouraging some individuals from accessing prevention options	• Person-centred approach improved willingness to engage in HIV prevention • Offering choices (PrEP vs. PEP, self-testing vs. provider testing) empowered users community-based service delivery increased accessibility	Explicitly aligned
3	Koss et al. [24]	Pre-exposure prophylaxis (PrEP) programme	Pre-exposure prophylaxis (PrEP)	• Rapid or same-day PrEP initiation • Flexible service delivery (clinic-based or community-based follow-ups) • Enhanced counselling for individuals at elevated HIV risk • Universal HIV testing with linkage to prevention and treatment	• HIV incidence was 74% lower among PrEP initiators compared to matched controls prior to PrEP availability • Among women, HIV incidence was reduced by 76%, while among men, incidence was 40% lower but not statistically significant • 79% of PrEP initiators engaged in at least one follow-up visit, and 61% self-reported adherence at least once • Rapid PrEP initiation and flexible service delivery can significantly reduce HIV incidence in generalised epidemic settings	• Low PrEP uptake due to stigma and misconceptions • Adherence challenges, particularly among younger populations • Limited awareness of PrEP benefits in rural communities	• Community-based service delivery improved accessibility • Flexible follow-up options encouraged retention in the program • Enhanced counselling helped individuals understand their HIV risk and prevention options	Community-based model with implicit/unclear PCC elements
4	Mataboge et al. [25]	Pre-exposure prophylaxis (PrEP) programme with decentralised service delivery	Pre-exposure prophylaxis (PrEP)	• Offering PrEP at non-traditional service points (pharmacies, schools, recreational spaces) • Integrated services combining PrEP with sexual and reproductive health (SRH) services • Choice of service locations to improve accessibility • Community engagement to reduce stigma and improve uptake	• Participants favoured decentralised and simplified PrEP service delivery, preferring health facilities, pharmacies, and educational institutions as service points • Long-acting Cabotegravir was preferred over the Dapivirine Vaginal Ring due to concerns about side effects, efficacy, and comfort • Recreational spaces were preferred for health information dissemination, particularly among adolescent boys and young men • The study suggests that expanding PrEP access beyond traditional clinics could improve uptake and engagement	• Healthcare provider attitudes and lack of privacy at clinics • Stigma and misconceptions about PrEP use • Long clinic waiting times and distance to facilities	• Decentralised service points improved accessibility • Integrated services reduced the burden of multiple clinic visits • Community engagement helped normalise PrEP use	Explicitly aligned
**5**	Williams et al. [26]^*^	Automated medication dispensing system (AMDS) for ART and Non communicable disease (NCD) medications	Automated medication dispensing system (AMDS) for ART and NCD medications	• Convenient access to ART and NCD medications without long waiting times • Non-stigmatising medication pickup process • Automated reminders via SMS for medication collection • Flexible pickup schedules to accommodate work and personal commitments • Integration with other health services to streamline care	• The AMDS was found to be feasible and acceptable among clients and healthcare workers • Phased introduction of medication classes starting with ART was effective • Improved client-centred messaging and communication increased system utilization • Consistent power supply and internet connectivity were critical for successful implementation	• Network connectivity issues affected system reliability • Limited awareness and messaging clarity led to confusion among clients • Power supply fluctuations disrupted system operations	• Stakeholder engagement ensured smooth implementation • Training of healthcare workers improved system adoption • Community involvement increased acceptance and utilization	Facility-based model with implicit/unclear PCC elements
6	Kabami et al. [27]	Dynamic Choice HIV Prevention (DCP) model	Pre-exposure prophylaxis (PrEP) or post-exposure prophylaxis (PEP)	• Choice of prevention product • Choice of service delivery location • Choice of HIV testing modality • Flexible refill duration of 1–3 months based on personal preference • 24/7 mobile-phone access to clinicians for support and adherence counselling	• PrEP uptake was highest in Antenatal Clinic (ANC) (100%), followed by OPD (86%) and community (50%) • PEP uptake was highest in the community setting (23%) compared to OPD (11%) and ANC (3%) • Biomedical prevention coverage was highest in ANC (80%), followed by OPD (60%) and community (32%) • Self-testing preference increased over time, from 38% at baseline to 58% at week 24	• Stigma and misconceptions about PrEP and PEP use • Limited awareness of HIV prevention options in rural communities • Concerns about privacy and confidentiality when accessing services	• Structured choice model empowered individuals to select preferred prevention options • Community-based service delivery improved accessibility • Flexible follow-up options encouraged retention in care	Explicitly Aligned
7	Kakande et al. [28]	Dynamic Choice HIV Prevention (DCP) model	Pre-exposure prophylaxis (PrEP) or post-exposure prophylaxis (PEP)	• Structured choice in selecting PrEP or PEP service location and testing modality • Ability to switch prevention • Personalised support plans addressing individual barriers • 24/7 phone access to a clinician for support	• Biomedical prevention coverage was significantly higher in the intervention group (28.0%) compared to the control group (0.5%) • During periods of self-reported HIV risk, coverage was 36.6% in the intervention group versus 0.9% in the control group • Self-testing preference increased from 52% at baseline to 71% at week 48 • 98% of intervention participants chose out-of-facility service delivery demonstrating strong preference for community-based care	Stigma and misconceptions about PrEP and PEP use • Limited awareness of HIV prevention options in rural communities • Concerns about privacy and confidentiality when accessing services	Structured choice model empowered individuals to select preferred prevention options • Community-based service delivery improved accessibility • Flexible follow-up options encouraged retention in care	Explicitly aligned
8	Jani et al. [29]	Psychosocial counselling aimed at reducing mental health problems and improving HIV-related outcomes	• Individual counselling • Group therapy sessions • Creative arts therapy (music, drama, art) • HIV education and safer sex counselling • Referral to sexual health services for HIV testing	• Tailored counselling based on individual needs • Community involvement through local service organisations • Empowerment strategies focused on decision-making and self-efficacy • Choice of intervention format (individual vs. group therapy)	• Significant improvements in knowledge and uptake of HIV prevention services among both male and female adolescents • Among female participants, aggressive behaviour decreased by 60% and overall mental health problems reduced by 50% • HIV knowledge, knowledge of where to get tested and actual HIV testing rates all increased among both genders, though males exhibited higher increases, especially in HIV testing uptake (630%) and sexual health service usage (220%) • Mental health benefits were observed only in female adolescents, highlighting the need for gender-specific intervention strategies	• Male adolescents, due to their street-based lifestyle, exhibited higher baseline mental health problems that were harder to address in a short intervention period • Female adolescents faced gender-based disadvantages, including lower education levels and increased vulnerability to abuse	• Community-based mentoring provided adolescents with structured support systems • Familiarity with service providers increased trust and engagement, particularly among male adolescents • Referral pathways to health services enabled increased HIV testing rates	Explicitly aligned
9	Zewdie et al. [30]	Pre-exposure prophylaxis (PrEP) programme	Differentiated PrEP delivery model incorporating • Direct-to-pharmacy PrEP refill visits • Client HIV self-testing (HIVST) • Pharmacy-led rapid risk assessment and dispensing	• Reduction of time spent in clinic, improving convenience • HIV self-testing option for greater autonomy • Pharmacist-led risk assessments, simplifying provider interactions • Enhanced privacy and reduced stigma concerns through streamlined processes	• The intervention significantly reduced total clinic visit time by more than one-third (from a median of 51 min to 33 min) • PrEP continuation rates were higher at intervention clinics (45% vs. 33% at month 1, 34% vs. 25% at month 3, and 23% vs. 16% at month 6) • HIV self-testing was highly acceptable, with 98% completion rates at month 1 and 94% at month 3 • PrEP adherence remained high, with detectable tenofovir concentrations in 85% of sampled clients	• Stigma concerns related to PrEP delivery within HIV care clinics • Limited infrastructure to support pharmacy-only PrEP services in some settings • High mobility of clients may affect long-term PrEP adherence	• Streamlined clinic visits reduced client burden and improved satisfaction • HIV Self Testing (HIVST) provided autonomy, reducing reliance on provider-led testing • Pharmacist-led assessments improved efficiency and engagement	Facility-based model with implicit/unclear PCC elements
10	Nkolo et al. [31]*	Differentiated Antiretroviral Therapy (DART) model	• Facility-Based Individual Management • Facility-Based Group • Fast-Track Drug Refills • Community Client-Led ART Delivery • Community Drug Distribution Point • Community Pharmacy	• Choice of care with clients selecting preferred ART delivery approaches • Tailored service delivery with less-intensive models for stable clients, more intensive care for clinically unstable individuals • Community engagement with community-led ART delivery improving accessibility • Autonomy and empowerment thus enabling self-directed ART management	• Clients in their preferred DART model had better clinical outcomes • Viral suppression was higher among clients in preferred models (85%) vs. non-preferred (68%) • Missed appointments were lower (29% vs. 40%) among those in preferred models • Demonstrates that person-centred, preference-aligned care improves treatment continuity and virological outcomes	• Mismatch between client preferences and available models such that 25% of clients were not receiving ART through their preferred model • High enrolment in facility-based individual management despite client preference for community-based models • Limited health system flexibility in adjusting ART delivery models based on evolving client needs	• Availability of community-based models • Flexibility of service delivery and multiple model options • Use of a structured preference tool for routine care	Explicitly aligned
11	Kamya et al. [32]	Dynamic Choice HIV Prevention model	Dynamic choice HIV prevention model incorporating • Oral pre-exposure prophylaxis (PrEP) • Post-exposure prophylaxis (PEP) • Cabotegravir long-acting injectable	• Choice-based model where participants could select and switch between prevention methods over time • Personalised counselling with structured assessment of barriers to adherence with tailored solutions • Community engagement with services delivered at local clinics and by community health workers • Accessibility enhancements with use of mobile health worker support and bridging oral PrEP for those unable to attend injection appointments	• Biomedical HIV prevention coverage was significantly higher in the intervention group (69.7%) compared to the control group (13.3%) • Cabotegravir long-acting injectable uptake was high, with 56% of intervention participants using it • HIV incidence was lower in the intervention group—zero new infections compared to seven infections in the control group over 48 weeks • Participants frequently switched between prevention methods, indicating the value of flexibility	• Adherence issues with oral PrEP due to Stigma and difficulties maintaining daily pill routines • Injection access barriers • Rural healthcare limitations	• Integrated services at community clinics improved accessibility • Mobile health support helped participants engage remotely • Flexible switching model allowed participants to modify their prevention choices as needed	Explicitly aligned
12	Cowan et al. [33]	Risk-differentiated, peer-led HIV prevention and care model	• Pre-exposure prophylaxis (PrEP) • Referral to antiretroviral therapy (ART) services -Contraception and condom provision • Syndromic management of sexually transmitted infections (STIs) • Health education and legal advice • Peer support programmes	• Shared decision-making through peer-led support • Tailored services based on risk levels • Choice of methods for HIV prevention • Strong community involvement via self-help groups • Empowerment strategies to build resilience	• There was no overall effect of the intervention on combined risk of HIV transmission and acquisition • Improved viral suppression in women living with HIV (93.5% in intervention vs. 88.8% in usual care) • PrEP uptake remained low, and effective engagement with the HIV prevention cascade was poor • Risk of HIV transmission among women living with HIV was significantly reduced (5.8% in intervention vs. 10.4% in usual care) • No significant difference in HIV acquisition risk among HIV-negative women	• Low adherence to oral PrEP among HIV-negative sex workers • Structural barriers to consistent condom use • Social stigma and mobility of sex workers, affecting retention	• Peer-led engagement improved ART uptake among HIV-positive women • Community-based support reduced treatment gaps for sex workers	Explicitly aligned

^$^ The Dynamic Choice HIV Prevention (DCP) model is a person-centred service delivery approach that enables individuals to tailor their HIV prevention experience across four domains: product type (e.g., PrEP or PEP), service location (clinic or community-based), testing modality (self-testing or provider-administered), and refill duration. It also includes 24/7 mobile access to clinicians, supporting autonomy, accessibility, and sustained engagement in care

* These treatment-focused studies were retained because they either (a) demonstrate treatment-as-prevention effects relevant to HIV prevention or (b) illustrate patient-centred service design features transferable to prevention interventions

### Person-centred care features

Across the 12 studies, a range of approaches were documented, all emphasising personalised service delivery, accessibility enhancements, shared decision-making, community involvement, and empowerment strategies (see Table 3). Of the included studies, nine explicitly described PCC features, while three primarily described decentralised, facility or community-based delivery models with implicit or unclear PCC principles. In four studies, choice-based prevention models were implemented, allowing participants to select preferred HIV prevention or ART service methods [23, 27, 28, 32]. Five studies incorporated conversational and structured counselling approaches as a key feature in HIV prevention [22, 23, 25, 28, 29]. Community-based HIV service delivery was featured in eight studies, with mobile health clinics, pharmacy-based PrEP distribution, and peer-led outreach programmes improving accessibility [23, 25–28, 30, 32, 33]. Empowerment strategies, such as peer mentoring, inclusion of personalised support plans and structured risk assessments, were recorded in four studies [27–29, 32]. Thematic analysis of the 12 studies revealed five key pillars influencing service delivery: personalised choice, accessibility, community support, cultural sensitivity and stigma reduction. The results are depicted in Table 4.

**Table 4:** Thematic analysis of person centred features of prevention services

Theme	Description/examples	Studies
Personalised choice	Allowing participants to select preferred HIV prevention or ART service models ensuring flexibility in care	• In Uganda, among 6376 ART clients, (4043/4623, 85%) viral suppression was observed in those receiving care via their preferred model, compared with (1007/1482, 68% ) in non-preferred models [31]
Accessibility and decentralisation	Moving HIV services beyond traditional clinics to pharmacies, schools, community centres, and mobile health platforms to improve reach	• In Kenya, a direct-to-pharmacy PrEP refill system reduced median clinic visit time from 51 min to 33 min, with higher PrEP continuation rates (118/264, 45%) at month 1, (91/264, 34%) at month 3, (61/264, 23%) at month 6 [30] • In South Africa, 109 participants engaged with a decentralised PrEP service, preferring pharmacies, schools, and recreational spaces over clinics [25]
Peer-led & community-based support	Empowering individuals through community engagement, peer mentoring, and support groups to enhance adherence and trust	In Zimbabwe, 4,268 female sex workers participated in a risk-differentiated, peer-led prevention model, leading to viral suppression rates of (863/931, 93.5%) vs. (927/1042, 88.8%) in standard care [33] • In Ethiopia, among 730 adolescents, male participants increased HIV testing rates by 630% and sexual health service utilisation by 220% through community mentoring programmes [29]
Stigma reduction & psychological safety	Non-judgemental provider interactions, self-testing options, and confidentiality measures to encourage engagement	• In Kenya, among 38 AGYW, higher PrEP adherence was observed when providers used non-judgemental counselling [22] • In pharmacy-led PrEP models at Kenya, (115/118, 98%) of participants completed HIV self-testing at month 1, (60/91, 94%) at month 3, reinforcing privacy and autonomy [30]
Cultural sensitivity & social context	Adapting services to respect cultural norms, gender roles, and local perceptions of HIV prevention	• In Kenya, providers used friendly, conversational approaches to build rapport and improve trust among young women, who often face stigma for seeking PrEP [22] • In Ethiopia, gender-based counselling approaches were emphasised, as male and female adolescents exhibited different levels of engagement with mental health and HIV testing services [29] • In Zimbabwe, interventions accounted for mobility patterns of sex workers, adjusting service delivery strategies to match their movement cycles and work environments [33]

### Outcomes and effectiveness

A total of six studies reported an increase in HIV prevention service uptake following intervention implementation, highlighting the impact of tailored, person-centred strategies in sub-Saharan Africa [23, 27–30, 32]. These studies documented significant improvements in PrEP continuation and adherence, with structured service delivery models showing higher engagement rates over time. In Kenya, the direct-to-pharmacy PrEP refill system led to (118/264) 45% retention at month 1, (91/264) 34% at month 3, and (61/264) 23% at month 6, demonstrating the advantages of streamlined access to prevention services [30]. Community-driven interventions targeting female sex workers and adolescent groups were also effective in improving adherence and health outcomes. In Zimbabwe, a risk-differentiated peer-led intervention enhanced viral suppression among HIV-positive sex workers, reaching (863/931) 93.5% compared with (927/1042) 88.8% in standard care [33]. Meanwhile, Ethiopia’s psychosocial counselling intervention led to a 630% increase in HIV testing among male adolescents and a 220% increase in sexual health service utilisation. For female adolescents, the same intervention resulted in a 60% reduction in aggressive behaviour and a 50% decrease in mental health problems, underscoring the broader benefits of integrated mental health and HIV prevention services [29]. Among studies evaluating ART adherence, differentiated antiretroviral therapy models demonstrated substantial improvements in viral suppression rates. In Uganda, ART clients receiving care through their preferred service model exhibited a viral suppression rate of (4043/4623) 85%, compared with (1007/1482) 68% in non-preferred models [31]. The adoption of HIV self-testing as part of differentiated prevention services also showed measurable improvements in client engagement. In Kenya, pharmacy-based self-testing models achieved completion rates of (115/118) 98% at month 1 and (69/91) 94% at month 3, reflecting strong acceptance among participants [30]. Biomedical prevention coverage, a key indicator of intervention success, showed marked improvements across structured service delivery models. In Kenya and Uganda, biomedical prevention coverage reached 47.5% in structured prevention choice models, compared with 18.3% in standard-care models [23]. In community-based dynamic prevention models, coverage was (57/202) 28.0% in intervention groups vs. (1/211) 0.5% in control groups, showcasing the effectiveness of decentralised service delivery [28].

### Barriers and facilitators to implementation

Stigma was the most frequent barrier reported in nine studies, manifesting as anticipated judgement, privacy concerns, and visibility of service use; these reduced PrEP initiation/continuation among AGYW and sex workers [22, 23, 25–28, 30, 32, 33]. Healthcare accessibility challenges were noted in six studies [23, 25, 26, 28, 30, 31], where logistical constraints such as long waiting times, limited clinic availability, and distance to service points discouraged service uptake. Adherence challenges were reported in five studies [23, 24, 27, 28, 32], particularly for interventions involving daily PrEP regimens. Structural and policy-related barriers were documented in six studies [25–27, 31–33], emphasizing systemic limitations in scaling HIV prevention services.

Several person-centred facilitators significantly improved HIV prevention engagement, adherence, and uptake. Community-based service delivery was reported as an enabler in nine studies [23, 25–30, 32, 33], particularly where peer-led models were integrated into prevention services. Decentralised service delivery was noted as a facilitator in four studies [25, 26, 30, 31], particularly where HIV prevention services were offered beyond traditional clinics. Tailored counselling and trust-building strategies were cited as facilitators in six studies [22, 23, 25, 28, 29, 32], highlighting the role of individualised care in improving retention and adherence. Mobile health innovations helped reduce barriers to HIV service accessibility in two studies [27, 32], particularly in PrEP and ART delivery.

### Equity and intersectionality in PCC models

Across the 12 included studies, few explicitly reported on equity dimensions. Some interventions targeted adolescents (particularly adolescent girls and young women) but rarely included adolescent boys. Engagement with sexual minorities and key populations (e.g., MSM, sex workers) was explicitly reported in two studies [25, 33], while older adults were largely absent. Geographic inequities were noted, with interventions concentrated in Eastern and Southern Africa. Very few studies presented outcomes stratified by subgroup (e.g., uptake by age or gender), limiting insights into how PCC models perform across diverse populations.

### Integration of non-HIV services

One (1) of the 12 included studies [33] described integration of non-HIV services into HIV prevention delivery. Integrated components included sexual and reproductive health/contraception (*n* = 1). Other components such mental health support, tuberculosis screening or linkage to TB care, and gender-based violence (GBV) screening or referral were not part of any included studies. Where integration was described, authors did not report on acceptability and convenience and failed to measure integrated service outcomes separately (e.g., contraception uptake).

## Discussion

This scoping review found that person-centred HIV prevention approaches in sub-Saharan Africa were present in Eastern and Southern Africa (notably Kenya, Uganda, South Africa, Zimbabwe, Eswatini and Ethiopia) and typically combined service flexibility (choice of method or setting), decentralised delivery, structured counselling, and peer/community engagement. By contrast, no studies were identified from West and Central Africa (WCA). This gap likely reflects a combination of methodological and structural factors. Methodologically, our search approach may have under-captured WCA work, including community-led models documented in reports, policy briefs, or French/Portuguese journals that are less likely to be indexed. Structurally, disparities in research funding, infrastructure, and publication pathways in low and middle income countries may reduce the visibility of WCA innovations in bibliographic databases, as noted by Nkhoma in 2022 [34]. Taken together, these factors suggest our map likely under-represents WCA activity, and the true geographic distribution of person-centred HIV prevention may be more even than our included studies suggest.

### Design flexibility, user choice, uptake and retention

Several studies illustrated how design flexibility enhances uptake. In Kenya, a differentiated PrEP model reduced clinic visit time and achieved strong adherence through pharmacy refills and HIV self-testing [30]. In Uganda, nearly all participants in a dynamic choice model selected out-of-facility care, with growing preference for self-testing [28]. Similar effects were observed in Nigeria and Ethiopia [35, 36]. These examples show how structured choice and decentralisation may improve both uptake and product retention thus facilitating HIV prevention. In contrast, the limited documentation of PCC models in West and Central Africa may reflect broader inequities in health system capacity and research visibility, as noted in other PCC related studies [34]. This regional disparity likely stems from persistent investment gaps, raising critical questions about who benefits from person-centred innovation and why geographic scaling remains uneven. User choice was also central to many interventions. Clients could select between oral and injectable PrEP [32], opt for facility-based or self-testing, and access services through schools, pharmacies, or mobile units. Such design flexibility aligns with PCC principles identified in broader HIV research across sub-Saharan Africa [37].

### Trust, peers and relational safety as core mechanisms

Beyond design, trust emerged as a critical enabler of success. Peer-led, community-embedded models were particularly effective among adolescent girls and young women, sex workers, and people living in stigmatised environments. In Uganda, clients receiving ART through community-led models achieved higher viral suppression when care aligned with their preferences indirectly promoting secondary HIV prevention [31]. Similar improvements were reported among clients receiving decentralised PrEP delivery in South Africa [25]. These findings underscore the importance of safety in relationships, peer support, and integrating community members formally into health systems to promote sustainability [38–40].

### Equity and intersectionality—gaps and missed opportunities

Despite the overall orientation towards flexibility, equity gaps remain. Most interventions focused on AGYW, with limited attention to adolescent boys, older adults, or rural communities. This narrow emphasis risks perpetuating inequities and undermines the broader goal of inclusive prevention. While programmes targeting AGYW showed positive outcomes—such as trusted provider relationships, youth-friendly counselling, and empowerment strategies [22]—similar benefits were observed when adolescent boys received psychosocial support and tailored outreach [29]. Yet such opportunities were often missed. In Burundi, intersecting barriers of age, geography, and gender hindered HIV testing among adolescents [41]. A systematic review further highlighted that older adults and people in low-resource settings are routinely excluded from PCC models, leaving them at disproportionate risk of neglect [34]. Conversely, inclusive designs—such as flexible delivery models and community engagement—improved participation among marginalised groups, including adolescent boys and individuals facing mental health challenges [29]. These results reinforce the need to tailor services to age, identity, and socio-economic status in order to promote equity in HIV prevention [42]. Future interventions should therefore explicitly develop models that address the needs of older adults, adolescent boys, and sexual minorities such as transgender and non-binary people. Equity could be assessed more robustly through uptake and retention disaggregated by age, gender, geography, and key population status; acceptability and satisfaction measured across subgroups and inclusion of marginalised voices in co-design processes. Such approaches would enable PCC interventions to move beyond general accessibility and tackle intersectional inequities in HIV prevention.

### Stigma and psychological safety

Finally, stigma and lack of psychological safety remain potent but often unspoken barriers. Evidence from multiple HIV self-testing (HIVST) studies shows that, although interventions may be physically accessible, uptake is often undermined by anticipated judgement, social repercussions, and fear of diagnosis—particularly among adolescents, men who have sex with men (MSM), and AGYW [43, 44]. In Kenya, HIVST provided autonomy and confidentiality, reducing reliance on providers and addressing fears of stigma [30]. Similar findings from the United States and South Africa revealed that home- or peer-delivered HIVST increased uptake by reducing visibility and providing emotional protection [45, 46]. By contrast, programmes that lacked supportive counselling or discretion in kit distribution saw markedly lower participation, as noted in a systematic review [47]. These findings highlight that stigma-sensitive design, peer engagement, and relational safety are not optional add-ons but maybe essential preconditions for equitable and sustained HIV prevention outcomes.

### Integration of Non HIV services as an element of PCC

The minimal reporting of non-HIV service integration in our included studies is an important finding. Person-centred care frameworks emphasise responsiveness to the whole person, which frequently requires linking HIV prevention to other health needs (e.g., family planning, mental health etc.). The observed focus on HIV-centred delivery suggests missed opportunities to provide holistic care that could increase convenience and address intersecting health needs—particularly for populations like AGYW, who often require sexual and reproductive services alongside HIV prevention. We therefore highlight integrated service delivery as a priority research and programmatic gap: future PCC interventions should proactively embed and measure non-HIV components and report their outcomes.

### Recommendations for implementing person-centred services

Taken together, these findings affirm that the most effective HIV prevention services are those co-created with users, respect individual preferences, and integrate psycho-social as well as biomedical support. Key steps that can be taken at various levels include:

#### Ministries of health

1. Scale up decentralised and community-based delivery models, reflecting evidence that these approaches improved uptake and retention in several studies.
2. Integrate psycho-social and stigma-sensitive components into national prevention guidelines, consistent with findings that relational safety and trust facilitated engagement.
3. Prioritise equity by expanding PCC approaches beyond AGYW to underserved groups identified as absent in the current evidence base.

#### Program designers and donors

1. Adopt structured choice models where appropriate, aligning with evidence that user autonomy supported sustained engagement.
2. Strengthen provider training to promote shared decision-making and non-judgmental communication, as highlighted across multiple studies.
3. Invest in implementation research to evaluate scalability of PCC models across West and Central Africa, where evidence gaps were most apparent.
4. Support integrated service models, addressing the lack of non-HIV service linkage noted across the included studies.

#### Community based organisations

1. Expand peer-supported interventions in populations where benefits were demonstrated (e.g., adolescents, sex workers).
2. Co-design services with priority communities, ensuring models reflect local preferences and reduce contextual barriers such as stigma.

## Limitations

This review provides a broad overview of person-centred HIV prevention in sub-Saharan Africa, but several limitations apply. First, studies were unevenly distributed, with Anglophone countries like Kenya, Uganda, and South Africa dominating; Francophone regions in Central and West Africa were under-represented, limiting regional balance. Second, while some groups (e.g., AGYW, MSM, university students) were covered, others—such as transgender people, people who inject drugs (PWID), older adults, and those with disabilities—were largely absent, reducing generalisability. Third, the included studies varied widely in design, often lacking standardised outcome measures and clear PCC frameworks, making comparisons difficult. Lastly, by focusing exclusively on peer-reviewed literature, the review may have missed valuable insights from grey literature and community-based reports, especially those reflecting informal or under-resourced settings. Future reviews should systematically integrate grey literature and non-English sources to improve representativeness and coverage. Nonetheless, the findings of the review are still useful in informing policy due to the comprehensive and systematic mapping of person-centred HIV prevention interventions across sub-Saharan Africa, revealing regional trends, population-specific insights, and critical implementation gaps that can inform future research and policy.

## Conclusion

This scoping review identified several person-centred HIV prevention models used in sub-Saharan Africa, including community-based and decentralised delivery, structured choice models, peer-supported interventions, psychosocial support approaches, and differentiated service delivery. These models were associated with improved uptake, engagement, retention, and user satisfaction, with some studies reporting enhanced adherence and viral suppression. Key facilitators included shared decision-making, trust-building relationships, decentralised access, and stigma-sensitive counselling. Common barriers were structural constraints, stigma, limited integration with broader health services, and geographical inequities in availability. Overall, person-centred strategies show promise but remain context-specific, with uneven implementation across populations and settings. Strengthening inclusivity, addressing structural barriers, and integrating user preferences will be essential for developing more equitable and effective person-centred HIV prevention programmes.

## Supplementary Information

Below is the link to the electronic supplementary material.

Supplementary Material 1.

Supplementary Material 2.

Supplementary Material 3.

## Data Availability

All data generated or analysed during this study are included in this published article [and its supplementary information files].
