# Supplementary material for: Person-centred HIV prevention services in sub-Saharan Africa: a scoping review
Source: AIDS Res Ther. 2026 Feb 5;23:33. doi: 10.1186/s12981-025-00839-0 (PMC12964596; doi:10.1186/s12981-025-00839-0)
Supplement: Supplementary file 1 — Supplementary Material 1. [file 12981_2025_839_MOESM1_ESM.docx]

Add Title Here

# Authors

First author name^1^ Second author name^2^ Third author name^3^ Fourth author name^4^ Fifth author name^5^ Sixth author name^6^

1. Affiliation
2. Affiliation
3. Affiliation
4. Affiliation
5. Affiliation
6. Affiliation

# Abstract

(Maximum - 250 words for Protocols/500 words for Reviews)

**Objective:** State an overarching review objective structured using the key components of the inclusion criteria (approximately one or two sentences). e.g. The objective of this scoping review is to understand the extent and type of evidence in relation to (insert field).

**Introduction:** Briefly describe the rationale for the review in light of what is already known on the topic (approximately two to three sentences).

**Inclusion criteria:** Summarize the inclusion criteria using the participants, concept, and context (PCC framework) and highlight any relevant exclusions in paragraph format. Present the information in one to three sentences – NOT under individual subheadings.

**Methods:** List the key information sources searched/to be searched (those from which the majority of evidence sources were/will be located), the date (month/year) the search was conducted (for reviews only) and any search limits (e.g. language). Briefly describe the approach to study selection, data extraction, analysis of the evidence and presentation of the results. Briefly describe any notable deviations to the methodological approach taken (for reviews only).

**Results (For Reviews ONLY):** The bulk of the abstract should be reserved to convey the main results of the review in relation to the objective/question. Report the number and type of included evidence as well as any pertinent study characteristics.

**Conclusions (For Reviews ONLY):** Provide a conclusion based on a general interpretation of the review findings in line with the review’s objective/s and any limitations of the review. Briefly convey key implications of the findings for practice and research (if any).

**Keywords:** List a maximum of five keywords in alphabetical order, separated by a semi-colon and a space. Ideally these words should be different to those that appear in the title and abstract. These are for the purposes of meta-data and indexing, and not related to the search strategy. 

# Introduction

 Describe the rationale for the review considering what is already known on the topic, including information that supports and justifies the selection of inclusion criteria. Key terms should be defined, and operational definitions narratively explained. Furthermore, provide some indication that there is evidence available that will meet your inclusion criteria. A rationale as to why a scoping review was the most appropriate method should also be provided. (Approximately 1000 words). For Reviews ONLY: The introduction must NOT be a verbatim reproduction of the introduction in the corresponding protocol.

**Subheading** (if required)

*Edit set text as appropriate:*

A preliminary search of MEDLINE, the Cochrane Database of Systematic Reviews and *JBI Evidence Synthesis* was conducted and no current or underway systematic reviews or scoping reviews on the topic were identified.

 If there are any existing systematic reviews/scoping reviews, it should be specified how the proposed review will differ.

Conclude with an overarching review objective, that captures and aligns with the core elements/PCC framework of the inclusion criteria (e.g. The objective of this scoping review is to assess the extent of the literature insert area/field.

# Review question

*Guidance for authors: Clearly state the review question or questions using the PCC framework (i.e. additional or sub-questions) that the review seeks to answer. The review question/s should clearly relate to the objective/s.*

# Inclusion criteria

### Participants

*Guidance for authors: Describe/define participants that will be included. Specific exclusion criteria based on any participant characteristics should also be stated.*

### Concept

*Guidance for authors: Describe and/or define the concept. Specific exclusion criteria based on any concept should also be stated.*

### Context

*Guidance for authors: Describe the context. Consider cultural/sub-cultural factors, geographic location, specific racial or gender-based interests or details about the specific setting. Specific exclusion criteria based on any context should also be stated.*

### Types of sources

*Edit set text as appropriate:*

This scoping review will consider both experimental and quasi-experimental study designs including randomized controlled trials, non-randomized controlled trials, before and after studies and interrupted time-series studies. In addition, analytical observational studies including prospective and retrospective cohort studies, case-control studies and analytical cross-sectional studies will be considered for inclusion. This review will also consider descriptive observational study designs including case series, individual case reports and descriptive cross-sectional studies for inclusion.

Qualitative studies will also be considered that focus on qualitative data including, but not limited to, designs such as phenomenology, grounded theory, ethnography, qualitative description, action research and feminist research.

In addition, systematic reviews that meet the inclusion criteria will also be considered, depending on the research question.

Text and opinion papers will also be considered for inclusion in this scoping review.

# Methods

The proposed scoping review will be conducted in accordance with the JBI methodology for scoping reviews.  insert a superscript citation to the JBI Manual for Evidence Synthesis or the relevant chapter and add this to the reference lis  Note: if the review title has been registered, report the name of the registry (e.g. OSF).

FOR REVIEWS: This review was conducted in accordance with an *a priori* protocol insert superscript citation to the published or in press protocol and add it to the reference list. Any deviations from the protocol should be reported and justified in the appropriate section of the methods.

### Search strategy

*Edit set text as appropriate:*

The search strategy will aim to locate both published and unpublished studies. A three-step search strategy will be utilized in this review.  First an initial limited search of MEDLINE (PubMed) and CINAHL (EBSCO) change as appropriate was undertaken to identify articles on the topic. The text words contained in the titles and abstracts of relevant articles, and the index terms used to describe the articles were used to develop a full search strategy for report the name of the relevant databases/information sources*(see Appendix #*). The search strategy, including all identified keywords and index terms, will be adapted for each included database and/or information source. The reference list of all included sources of evidence will be screened for additional studies. *Specify the types of references examined (eg, references of studies included in the systematic review, or references of systematic reviews on the same or similar topic*. *Modify if other processes are to be used, eg, Report details of any tools to identify keywords or index terms, any filters, any validation processes, whether search strategy will be peer reviewed.]*

 Studies published in any language change as appropriate will be included. Studies published since insert date will be included as

justify date range and any language limitations

*List all information sources (eg, electronic databases, conference proceedings, websites, search engines or other online sources, contact with study authors etc.* The databases to be searched include insert databases with platforms as appropriate. Sources of unpublished studies and gray literature to be searched include insert text.

### Study/Source of evidence selection

*Edit set text as appropriate:*

Following the search, all identified citations will be collated and uploaded into (insert the name of the bibliographic software or citation management system e.g. EndNote insert version /year (Clarivate Analytics, PA, USA) and duplicates removed. Following a pilot test, titles and abstracts will then be screened by two or more independent reviewers for assessment against the inclusion criteria for the review. Potentially relevant sources will be retrieved in full and their citation details imported into the JBI System for the Unified Management, Assessment and Review of Information (JBI SUMARI) (JBI, Adelaide, Australia). insert citation to JBI SUMARI paper and add it to the reference list, eg, <Munn Z, Aromataris E, Tufanaru C, Stern C, Porritt K, Farrow J. The development of software to support multiple systematic review types: the Joanna Briggs Institute System for the Unified Management, Assessment and Review of Information (JBI SUMARI). Int J Evid Based Healthc. 2019;17(1):36-43.>The full text of selected citations will be assessed in detail against the inclusion criteria by two or more independent reviewers. Reasons for exclusion of sources of evidence at full text that do not meet the inclusion criteria will be recorded and reported in the scoping review. Any disagreements that arise between the reviewers at each stage of the selection process will be resolved through discussion, or with an additional reviewer/s. The results of the search and the study inclusion process will be reported in full in the final scoping review and presented in a PRISMA flow diagram

(insert citation to PRISMA and include in the reference list)  .

### Data extraction

*Edit set text as appropriate:*

Data will be extracted from papers included in the scoping review by two or more independent reviewers using a data extraction tool developed by the reviewers. cite the tool to be used or append the data extraction tool if an existing tool has been modified or a new tool developed any modifications to existing tools should be described in the text  The data extracted will include specific details about the participants, concept, context, study methods and key findings relevant to the review question/s.

*Guidance for authors: Discuss the planned piloting of the draft extraction form in the protocol, and how this was conducted in the review.*

*Edit set text as appropriate:*

A draft extraction form is provided (see Appendix XX). The draft data extraction tool will be modified and revised as necessary during the process of extracting data from each included evidence source. Modifications will be detailed in the scoping review. Any disagreements that arise between the reviewers will be resolved through discussion, or with an additional reviewer/s. If appropriate, authors of papers will be contacted to request missing or additional data, where required.

*Guidance for authors: Critical appraisal of individual sources of evidence: This is generally not required for scoping reviews. If it will be done, provide a rationale as to why and describe the methods, including tools which will be used.*

### Data analysis and presentation

*Guidance for authors: The evidence presented should directly respond to the review objective and question(s). The data is commonly presented graphically or in diagrammatic or tabular form. Preparation of the review protocol is the opportunity for authors to pilot and determine how to best present their data or map and provide detailed description for the reader. Insert information on data presentation/mapping techniques, if any.  A narrative summary will accompany the tabulated and/or charted results and will describe how the results relate to the reviews objective and question/s.*

# Acknowledgements

Insert the full names and precise contributions of individuals, or institutions, who have not already been listed as co-authors. Specify if this review is to contribute towards a degree award and for which author (initials). For further guidance, please refer to the *JBI Evidence Synthesis* manuscript style and preparation guidelines.

# Funding

Guidance for authors: Provide details on sources of funding for the review and explicitly describe the role of funders in the review process. For further guidance, please refer to the *JBI Evidence Synthesis* manuscript style and preparation guidelines.

# Declarations

Authors are invited to consider equity, diversity, and inclusion to acknowledge authors who work to improve diversity and inclusion in research and to encourage them going forward. Authors may also choose to highlight their experience related to their topic to further position them within the field (eg, an Indigenous author writing about issues directly related to Indigenous populations).

# Author contributions

Detail each author’s specific involvement in a manuscript, such as designing the analysis, contributing or collecting the data, performing the analysis or writing the manuscript, to increase the transparency of contributions.

# Conflicts of interest

Guidance for authors: Include a statement that describes a potential conflict of interest or any personal, financial, professional, or intellectual bias for any of the authors listed on the manuscript. If no conflict exists, include the following statement: There is no conflict of interest in this project. For further guidance, please refer to the *JBI Evidence Synthesis* manuscript style and preparation guidelines.

# References

# Appendices

### Appendix I: Search strategy

For protocols - present a full search strategy for at least one electronic database including planned limits, such that it can be reviewed and repeated. For systematic reviews all search strategies should be presented.

The search strategy should detail the following information: the name of the information source and the platform/service provider used to search the particular database, eg, CINAHL (via Ovid); all the search terms to be used (both keywords/text words and index terms should be included) and how they are to be combined using Boolean logic; the use of truncation and wildcards; all planned limits (date, language, etc.); and the number of records retrieved by the search.

Insert name of database (insert name of the platform/service provider), e.g. MEDLINE (Ovid)

Search conducted on

month/year

### Appendix II: Data extraction instrument

#Only append the JBI or non-JBI data extraction instrument if the standardized tool has been modified in any way, otherwise simply cite the tool used in the text. Any modifications made to the instrument should also be described in the text.#
