# Supplementary material for: Person-centred HIV prevention services in sub-Saharan Africa: a scoping review
Source: AIDS Res Ther. 2026 Feb 5;23:33. doi: 10.1186/s12981-025-00839-0 (PMC12964596; doi:10.1186/s12981-025-00839-0)
Supplement: Supplementary file 2 — Supplementary Material 2. [file 12981_2025_839_MOESM2_ESM.docx]

**Appendix 1 of Full search strings for PUBMED with strings**

**#1** "HIV prevention"[MeSH Terms]

**#2** "HIV prevention services"[All Fields]

**#3** ("HIV"[All Fields] AND "prevention"[All Fields])

**#4** #1 OR #2 OR #3

**Patient-centred care terms**

**#5** "patient-centred care"[MeSH Terms]

**#6** "person-centred"[All Fields]

**#7** "client-centred"[All Fields]

**#8** "patient focused"[All Fields]

**#9** #5 OR #6 OR #7 OR #8

**Geographic terms**

**#10** "Africa South of the Sahara"[MeSH Terms]

**#11** "sub-Saharan Africa"[All Fields]

**#12** #10 OR #11

**Final combination of strings**

**#13** - #4 AND #9 AND #12

Final search results - 22 studies

**Appendix 2: Sequential PubMed string with multilingual terms in Portuguese and French**

#1 "HIV prevention"[MeSH Terms] OR "HIV prevention services"[All Fields] OR (HIV[All Fields] AND prevention[All Fields])

#2 "prévention du VIH"[All Fields] OR "prévention du SIDA"[All Fields] OR (VIH[All Fields] AND prévention[All Fields])

#3 "prevenção do HIV"[All Fields] OR "prevenção da SIDA"[All Fields] OR (HIV[All Fields] AND prevenção[All Fields])

#4 #1 OR #2 OR #3

#5 "Patient-Centered Care"[MeSH Terms] OR "patient centered"[All Fields] OR "patient centred"[All Fields]

OR "person centered"[All Fields] OR "person centred"[All Fields]

OR "client centered"[All Fields] OR "client centred"[All Fields] OR "patient focused"[All Fields]

#6 "soins centrés sur le patient"[All Fields] OR "soins centrés sur la personne"[All Fields] OR "soins axés sur le patient"[All Fields]

#7 "cuidados centrados no paciente"[All Fields] OR "cuidados centrados na pessoa"[All Fields]

#8 #5 OR #6 OR #7

#9 "Africa South of the Sahara"[MeSH Terms] OR "sub-Saharan Africa"[All Fields]

OR "Afrique subsaharienne"[All Fields] OR "Afrique de l’Ouest"[All Fields] OR "Afrique centrale"[All Fields]

OR "África Subsaariana"[All Fields] OR "África Ocidental"[All Fields] OR "África Central"[All Fields]

**Final combination of strings**

#10 #4 AND #8 AND #9
