# Supplementary material for: Person-centred HIV prevention services in sub-Saharan Africa: a scoping review
Source: AIDS Res Ther. 2026 Feb 5;23:33. doi: 10.1186/s12981-025-00839-0 (PMC12964596; doi:10.1186/s12981-025-00839-0)
Supplement: Supplementary file 3 — Supplementary Material 3. [file 12981_2025_839_MOESM3_ESM.docx]

**Supplementary Material**

List of Included Studies in Scoping Review

1. Omollo V, Roche S, Mogaka F, Odoyo J, Barnabee G, Bukusi EA, et al. Provider–client rapport in pre-exposure prophylaxis delivery: a qualitative analysis of provider and client experiences of an implementation science project in Kenya. Sexual and reproductive health matters. 2022 Sep 28;30(1). Available at: https://doi.org/10.1080/26410397.2022.2095707
2. Koss CA, Ayieko J, Kabami J, Balzer LB, Kakande E, Sunday H, et al. Dynamic choice HIV prevention intervention at outpatient departments in rural Kenya and Uganda. AIDS [Internet]. 2023 Oct 19 [cited 2024 Sep 23];38(3):339–49. Available from: https://pubmed.ncbi.nlm.nih.gov/37861683/
3. Koss CA, Havlir DV, Ayieko J, Kwarisiima D, Kabami J, Chamie G, et al. HIV incidence after pre-exposure prophylaxis initiation among women and men at elevated HIV risk: A population-based study in rural Kenya and Uganda. PLoS Medicine [Internet]. 2021 Feb 9;18(2). Available from: https://www.ncbi.nlm.nih.gov/pmc/articles/PMC7872279/
4. Paballo Mataboge, Nzenze S, Nqaba Mthimkhulu, Mbali Mazibuko, Kutywayo A, Butler V, et al. Planning for decentralized, simplified prEP: Learnings from potential end users in Ga-Rankuwa, gauteng, South Africa. Frontiers in Reproductive Health. 2023 Jan 9;4. Available from: https://doi.org/10.3389/frph.2022.1081049
5. Williams V, Samson Haumba, Fikile Ngwenya-Ngcamphalala, Mafukidze A, Normusa Musarapasi, Hugben Byarugaba, et al. Implementation of the Automated Medication Dispensing System–Early Lessons From Eswatini. International Journal of Public Health [Internet]. 2023 Oct 12;68. Available from: https://doi.org/10.3389/ijph.2023.1606185
6. Kabami J, Kakande E, Chamie G, Balzer LB, Petersen M, Camlin CS, et al. Uptake of a patient‐centred dynamic choice model for HIV prevention in rural Kenya and Uganda: SEARCH SAPPHIRE study. Journal of the International AIDS Society. 2023 Jul 1;26(S1). Available at: https://doi.org/10.1002/jia2.26121
7. Kakande ER, Ayieko J, Sunday H, Biira E, Nyabuti M, Agengo G, et al. A community‐based dynamic choice model for HIV prevention improves PrEP and PEP coverage in rural Uganda and Kenya: a cluster randomized trial. Journal of the International AIDS Society. 2023 Dec 1;26(12). Available at: https://doi.org/10.1002/jia2.26195
8. Jani N, Vu L, Kay L, Habtamu K, Kalibala S. Reducing HIV-related risk and mental health problems through a client-centred psychosocial intervention for vulnerable adolescents in Addis Ababa, Ethiopia. Journal of the International AIDS Society. 2016 Jul;19:20832. Available at: https://doi.org/10.7448/IAS.19.5.20832
9. Zewdie KB, Ngure K, Mwangi M, Mwangi D, Maina S, Etyang L, et al. Effect of differentiated direct‐to‐pharmacy PrEP refill visits supported with client HIV self‐testing on clinic visit time and early PrEP continuation. Journal of the International AIDS Society. 2024 Mar 1;27(3). Available at:https://doi.org/10.1002/jia2.26222
10. Nkolo E, Jessica Clinkscales Ejike, Sensalire S, Ssali J, Immaculate Ddumba, Calnan J, et al. Clients in Uganda accessing preferred differentiated antiretroviral therapy models achieve higher viral suppression and are less likely to miss appointments: a cross‐sectional analysis. Journal of the International AIDS Society [Internet]. 2023 Jul 1 [cited 2023 Oct 5];26(S1). Available from: https://www.ncbi.nlm.nih.gov/pmc/articles/PMC10323312/
11. Kamya MR, Balzer LB, Ayieko J, Kabami J, Kakande E, Chamie G, et al. Dynamic choice HIV prevention with cabotegravir long-acting injectable in rural Uganda and Kenya: a randomised trial extension. The lancet HIV [Internet]. 2024 Nov;11(11):e736–45. Available from: https://pubmed.ncbi.nlm.nih.gov/39395424/
12. Cowan FM, Machingura F, Ali MS, Chabata ST, Takaruza A, Dirawo J, et al. A risk-differentiated, community-led intervention to strengthen uptake and engagement with HIV prevention and care cascades among female sex workers in Zimbabwe (AMETHIST): a cluster randomised trial. The Lancet Global Health [Internet]. 2024 Sep [cited 2024 Sep 19];12(9):e1424–35. Available from: https://pubmed.ncbi.nlm.nih.gov/39151978/
